# Correspondence

**Published:** 1902-07

**Authors:** E. G. Ferguson

**Affiliations:** Macon, Ga.


### CORRESPONDENCE.

*Editor Detroit Medical Journal:*

Your editorial is without provocation and of such a foreign character to the subject you are supposed to discuss that I look upon it as a piece of steeple-eared work. In the first place, the subject I wrote of was the traits of a nigger, and as fully as in my power and scope of observation, extending over a period of twenty-seven years, did I delineate his characteristics. 'Tis true I did not view him as a brother, as you in your comments did, but as a nigger, with traits peculiar to the race that I have these long years noted without in any way having a desire to hug or kiss the black brute. In their place, as cattle, I have the highest regard for them, and if left to me would emasculate all the males and ovariotomize all the females, so as to stop the breed, or send them to Michigan, where they would be properly cared for and treated like brothers and sisters by the Michiganders, where they could rape a few wives and daughters without the danger of being burnt at the stake, as well as steal all they could get their hands on. You think they are not properly treated here. They are looked upon as cattle, and very good ones at that, and are accordingly worked. A piece of advice: When they get there keep everything under lock and key; glue your shirt to your back and cement your pillowslips to the pillows and bed sheets to the bed, or you will lose them. Some smart fellow can devise a means by which to bottle up some of their effluvium in summer for winter use, so you will always be certain to have at least his characteristic odor with you. He might also save the clippings of wool and start a knitting factory for socks. In my article I did not say that any feature was a *sine qua non* to his usefulness, as you so flagrantly misrepresent me, but simply gave his traits or ethnology as they appear to all of us here, who are very much kinder to him than any one living North. I do not happen to be a Southerner, as you quote me, but am a

Canadian, and have made this my home for twenty-seven years, and all I have written about the race is nothing but their traits as they appear to me.

Inasmuch as you have assailed me in the manner in which you have, in justice to myself and journalistic courtesy due me, I ask space in your journal for this reply.

Yours, E. G. FERGUSON, M.D., Macon, Ga.

---

### DIETETIC APHORISMS FOR INFANT LIFE.

The following are the aphorisms which are offered :

1. Nature's way and Nature's food are the best. The possibility of the improvement of the quality or quantity of the mother's milk should always be considered before putting the child on artificial feeding.

2. We should do the best we can with what we have. Here Griffith protests against the adoption of any one fixed formula for infant feeding. The mixture must be made to meet the special requirements of the child.

3. Keep up with the times. What is unscientific will not pass muster. He gives formulas and equations of infant food, giving the most recent and from experience the most suitable.

4. Know what you want. We should know why we give this or that mixture to certain children and should not be lazy or too adherent to old ways.

5. Go slow and do not increase the strength of the milk too rapidly or introduce too much starchy food. The age of the child should not be the guide, but its general condition, especially its weight, should be.

6. Lastly, he mentions the starvation treatment, meaning by this the judicious temporary reduction in the amount and strength of the food given, to meet the necessities of the case.—*Jour. A. M. A.*

